# Hypertension control: results from the Diabetes Care Program of Nova Scotia registry and impact of changing clinical practice guidelines

**DOI:** 10.1186/1475-2840-4-11

**Published:** 2005-07-20

**Authors:** Cory Russell, Peggy Dunbar, Sonia Salisbury, Ingrid Sketris, George Kephart

**Affiliations:** 1 Department of Community Health and Epidemiology, Dalhousie University, Halifax, Canada; 2 Diabetes Care Program of Nova Scotia, Halifax, Canada; 3 College of Pharmacy, Dalhousie University, Halifax, Canada

## Abstract

**Background:**

The objective of this study was to determine the rate of blood pressure control according to 4 sets of Canadian guidelines published over a decade in patients with diabetes mellitus attending Diabetes Centres in the province of Nova Scotia.

**Methods:**

One hundred randomly selected charts from each of 13 Diabetes Centres audited between 1997 and 2001 were extracted from the Diabetes Care Program of Nova Scotia Registry. Multivariate logistic regression analyses examined the relationship between individual characteristics and self-reported antihypertensive use. Included were 1132 adults, mean age 63 years (48% male), with 9 years mean time since diagnosis of diabetes.

**Results:**

According to the 1992 guidelines, 63% of the patients and according to the 2003 guidelines, 84% of patients were above target blood pressure or receiving antihypertensive medications. Forty-seven percent of patients are considered to be hypertensive and not on treatment according to 2003 guidelines. The results of the multivariate analyses showed that the only factors independently associated with anti-hypertensive use was oral anti-hyperglycemic use.

**Conclusion:**

Hypertension is an additional risk factor in those with diabetes mellitus for macrovascular and microvascular complications. The health and budgetary impacts of addressing the treatment gap need to be further explored.

## Background

Achievement of target blood pressures in hypertensive patients is often difficult. In Halifax County, Nova Scotia, 57% of men and 42% of women with hypertension were not well controlled[1]. Adequate blood pressure control is of particular concern in patients with diabetes as hypertension increases morbidity and mortality associated with stroke and cardiovascular disease[2,3], as well as microvascular complications such as retinopathy and nephropathy[4]. Cardiovascular disease rates have been shown to be 2–4 times higher in diabetes than in matched non-diabetic populations[5,6].

A Canadian study reported 43% of people (age 18–74 years) had an optimal blood pressure (<120/80 mmHg), and of those with a diagnosis of hypertension, only 13% were below target (defined as 140/90 mm Hg). In this study, about 50% of patients with diabetes were hypertensive, and of these only 9% were under control[7]. An internal review at the Diabetes Care Program of Nova Scotia (DCPNS) from 1997–2001 showed that only 27.5% of a random selection of patients attending Diabetes Centres fell within the recommended target blood pressure for people with a diagnosis of diabetes (< 130/85 mm Hg) [8].

The United Kingdom Prospective Diabetes Study (UKPDS) emphasized the need for adequate blood pressure control in type 2 diabetes. The evidence suggested that good blood pressure control may be as important if not more important than blood glucose control in reduction of the cardiovascular complications[3,9,10]. Further, adequate blood pressure control in the UKPDS decreased risk for multiple diabetes end-points: 32% in deaths related to diabetes; 44% decreased risk of stroke; and a 34% reduction in risk for all macrovascular diseases, as well as a significantly decreased risk for other complications. [4] Clinical trials and epidemiologic studies have suggested the target blood pressure goal of <130/80 mmHg[11-14].

The treatment of hypertension in patients with diabetes has changed over the last decade. Studies and Clinical Practice Guidelines for the management of hypertension in patients with diabetes suggest lower blood pressure targets for diagnosis and control than for the general population[11,15-17].

This study determined the degree of blood pressure control in patients with diabetes according to four sets of Canadian Clinical Practice Guidelines published between 1992 and 2003[2,18-20] and described demographic and treatment variables associated with antihypertensive treatment.

## Methods

The cohort was selected as part of a DCPNS (Diabetes Care Program of Nova Scotia) internal audit of approximately 100 records from each of 13 Diabetes Centres between 1997 and 2001. All patients were referred to the Diabetes Centre following a diagnosis of diabetes by a physician. Information gathered included: age, gender, weight, blood pressure, duration of diabetes, serum creatinine, urinary protein and specific antihypertensive treatment regimens. The mercury sphygmomanometer was used for blood pressure measurement and recorded by nurses and averaged over all visits for all individuals. The nurses were aware of the correct procedure for obtaining a blood pressure measurement, and performed the procedure regularly. Eligibility criteria for the cohort included being a non-pregnant adult over the age of 19; a diagnosis of type 1 or 2 diabetes; a visit to the centre within 12 months of the audit date; and at least 15 months of followup.

The final cohort included 1132 subjects. The population consists of both genders (48% male), with an average age of 63 (SD 12). Over 95% of patients had type 2 diabetes, and the average length of time since diagnosis was 9.3 (SD 8) years previous. Average scores were obtained for most tests and attributes. Kidney function was estimated using both Couchoud cutpoints and the Cockroft-Gault formula [21,22] Drug information was reclassified using the WHO Anatomical Therapeutic Chemical (ATC) categories[23]. Prevalence of hypertension and trends in Clinical Practice Guidelines over time were determined.

Guidelines used for analysis included the following: 1992 Clinical Practice Guidelines for Treatment of Diabetes Mellitus – hypertension subcategory [18]; 1998 Clinical Practice Guidelines for the Management of Diabetes in Canada – hypertension subcategory [2]; 1999 Canadian Hypertension Society Recommendations for the Management of Hypertension – diabetes subcategory [19]; 2003 Canadian Hypertension Society Recommendations for the Management of Hypertension – diabetes subcategory[20].

Hypertension was defined using anti-hypertensive drug use and blood pressure records. Patients with any antihypertensive drug use and/or average blood pressure above the guideline cutpoints (systolic, diastolic, or both) were designated to be hypertensive. Rates and risk factors for hypertension were calculated for each specific guideline. Logistic regression was performed to determine predictors of antihypertensive treatment.

SAS version 8.2 (SAS Institute, Cary, NC, USA, 2001) was used for analysis.

## Results

**Figure 1:**
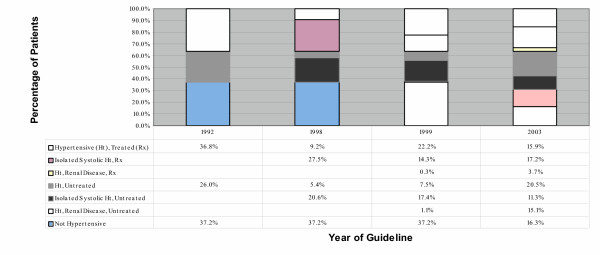
The effect of clinical practice guidlines changes on the percentage of Nova Scotia patients with diabetes classified as hypertensive.

The use of the 2003 guidelines (target blood pressure: systolic < 130 mmHg; diastolic < 80 mmHg) increased the percentage of patients not meeting target to 84% from 63% using 1992 guidelines (target blood pressure: systolic < 140 mmHg; diastolic <90 mmHg). Those considered to be hypertensive and not on treatment increased to 47% using the 2003 guidelines from 26% with the 1992 guidelines. (Figure 1); Clinical Practice Guidelines Effects on Nova Scotia Patients with Diabetes Classified as Hypertensive; Blank cells indicate that the category was not applicable for that guideline. The "Isolated Systolic" category in the 1999 and 2003 guidelines is used synonymously with the "Elderly" category used in the 1998 guidelines for data display purposes) Among all potential predictors of antihypertensive drug treatment related to the 2003 guidelines included in our database, only the patients receiving oral antihyperglycemics with or without insulin were more likely to be treated. (Table 1: Predictors of Treatment among Patients with Hypertension, 2003 Guidelines)

**Table 1:** Predictors of Treatment among Patients with Hypertension, 2003 Guidelines

Variable	Crude Odds Ratio (95% CI)	Adjusted Odds Ratio (95% CI)†
Age		
≤50	1.00	
51–60	1.17 (0.70–1.96)	
61–70	1.47 (0.91–2.37)	
≥71	1.42 (0.88–2.28)	

Gender		
Male	1.00	
Female	1.26 (0.97–1.63)	

Diabetes		
Type I	1.55 (0.75–3.18)	
Type II	1.00	

Treatment *		
Diet	1.00	1.00
Oral meds	1.89 (1.31–2.74)	1.89 (1.31–2.74)
Insulin	1.04 (0.77–1.41)	1.04 (0.77–1.41)
Insulin + Oral	2.48 (1.13–5.43)	2.48 (1.13–5.43)

Kidney Disease **		
None	1.00	
Mild	1.32 (0.90–1.94)	
Moderate	0.97 (0.65–1.45)	
Severe	1.23 (0.34–4.50)	
Failure	not valid	

Years Since Diagnosis		
≤5	1.00	
6–10	0.86 (0.62–1.19)	
≥11	1.32 (0.98–1.78)	

Years Since Referral		
≤3	1.00	
4–7	0.84 (0.61–1.17)	
≥8	0.94 (0.67–1.31)	

Weight (KG)		
≤55	1.00	
56–67	0.78 (0.35–1.71)	
68–90	0.87 (0.42–1.80)	
≥91	0.99 (0.47–2.06)	

* Oral meds mean taking only oral antihyperglycemic medication; Insulin + Oral means patients who are taking insulin plus oral antihyperglycemic medication.

** Presence and stage of kidney disease as measured using the Cockroft-Gault formula.

† Treatment was the only significant predictor, and therefore is not adjusted.

## Discussion

Many Nova Scotia patients with diabetes mellitus had uncontrolled blood pressure and were not receiving antihypertensive medication. Achieving control of high blood pressure may be more important for long-term outcomes than glycemic control[3,10]. The rates were similar to other studies where 54–58% were above target blood pressure and 22–28% were not receiving antihypertensive treatment [24,25]. These populations have a decreased prevalence of hypertension, yet a higher rate of treatment in those affected.

Changing Clinical Practice Guidelines affect the criteria for diagnosis, the treatment targets, the population to be treated and the type of treatment. Many patients with diabetes mellitus previously considered to be normotensive are now above the defined cutpoints. Adherence to the newer guidelines would result in more patients being treated and increased drug expenditures, but may lead to decreased overall health service utilization and improved patient outcomes. Further work will be needed to determine the rate of adoption of the newer guidelines and the facilitators and barriers to adoption. For example, it is unclear how well guidelines apply to patients above age 85 or the frail elderly.

This study is a population-based study in the real world. The study included cardiovascular risk factors, and documentation of kidney disease unlike many survey reports[26]. Drug data was recorded by patient self-report at each visit by Diabetes Centre personnel. The quality of the DCPNS Registry evolved over time, particularly the details related to antihypertensive drug therapy. Self-report has had good concordance with pharmacy claims data[27,28]. We were unable to determine how patients used the medications, if antihypertensive medications were used for hypertension or for another disease, any contraindications to therapy, the comorbid conditions, target organ damage, or response to previous antihypertensive therapy. Blood pressure measurements were part of routine care. Family history of cardiovascular disease, smoking, and lifestyle factors and the level of blood pressure at which treatment was started were not determined.

## Conclusion

Many patients with diabetes mellitus and hypertension were not treated according to guidelines, with 47% of the patients meeting the 2003 guidelines definitions of hypertension not being treated with antihypertensive medications. By reducing the cutpoints for defining hypertension, the proportion of people affected increased substantially. Specific risk factors determined may aid in identifying patients at high-risk for inadequate treatment. Patient and provider education, public health approaches, and health system changes are needed to address these issues. Further work is needed to determine the reasons for lack of control, approaches to improve control and long-term patient outcomes, and the budget impact and cost effectiveness of using the 2003 guidelines.

## Competing Interests

The author(s) declare that they have no competing interests.

## Authors' Contributions

Cory Russell was involved with the design of the study, analysis and interpretation of the data, drafting and editing the manuscript, and gave final approval of the manuscript. All other authors were involved with the design of the study, interpretation of the data, editing and revising the manuscript, and gave final approval of the manuscript to be published.

## Financial Support

*Cory Russell *was funded by the Drug Use Management and Policy Residency, a summer studentship that aims to build student and faculty understanding about how the creation of knowledge and dissemination of evidence is used by decision makers for drug used management and policy analysis. The Canadian Health Services Research Foundation, Canadian Institutes of Health Research and the Nova Scotia Health Research Foundation support this residency.

*Dr. Ingrid Sketris *holds a Chair funded by the Canadian Health Services Research Foundation(CHSRF)/Canadian Institutes for Health Research (CIHR) Chair in Health Services Research, co-sponsored by the Nova Scotia Health Research Foundation (NSHRF).

## Declaration of Business Interests/Disclaimer

The data used in this research was made available by the Diabetes Care Program of Nova Scotia. Any opinions expressed by the authors do not necessarily reflect the opinion of DCPNS.

Presented in part at the 2004 Canadian Association for Population Therapeutics conference, June 6–8 2004 in Winnipeg, Mb

## Note

The Effect of Clinical Practice Guidelines Changes on the Percentage of Nova Scotia Patients with Diabetes Classified as Hypertensive
